# Time-resolved x-ray absorption spectroscopy probe in ultrafast surface chemistry

**DOI:** 10.1063/4.0000289

**Published:** 2025-02-05

**Authors:** Anders Nilsson

**Affiliations:** Department of Physics, Stockholm University, 10691 Stockholm, Sweden and SUNCAT Center for Interface Science and Catalysis, SLAC National Accelerator Laboratory, 2575 Sand Hill Road, Menlo Park, California 94025, USA

## Abstract

To celebrate the scientific achievement of Jo Stöhr, I present here a personal account of the use of x-ray absorption spectroscopy to probe dynamics on surfaces using x-ray lasers. In particular, I will review the investigation of ultrafast processes in adsorbates on surfaces using an optical pump and an x-ray absorption spectroscopy probe. Here, it is shown that it is possible to gain insight into the effects of electronic excitations in metals on adsorbates as well as laser-induced vibrational motions. Furthermore, the ultrafast optical pump allows the detection of the CO precursor state in the desorption channel, species close to the transition state in CO oxidation, and the transient HCO intermediate during CO hydrogenation on Ru(0001).

## I. INTRODUCTION

**FIG. 1.**
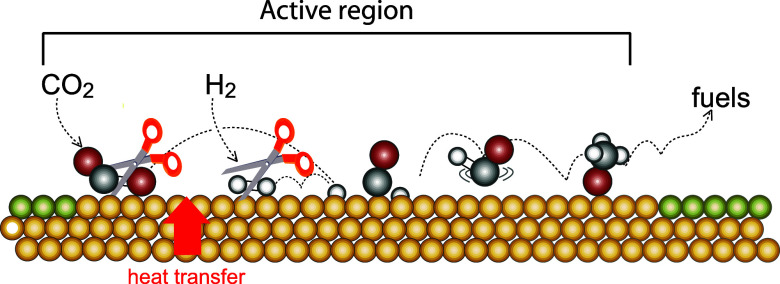
Catalytic reactions involving CO_2_ at the gas–solid interface. Scission of the adsorbate by thermal energy transfer is the key elementary process of all the system.

Most of the chemical processes involved in energy conversion and in the synthesis of base chemicals rely on catalytic chemical transformations at interfaces between solids and liquids or gases. These interfacial reactions include electro- or photo-catalytic processes for hydrogen production and CO_2_ conversion to fuels, enhanced fuel cell catalysts, and selective thermal heterogeneous catalytic processes for ammonia, methanol, higher alcohols, and hydrocarbons generation. Due to the importance of these processes, it is essential to gain a fundamental understanding of the factors that control the reaction mechanism, thereby the overall activity, selectivity, and stability of the catalytic material under various conditions, such as different temperatures and pressures. The surface chemical reactions relevant to catalysis proceed through several elementary reaction steps that control the overall reaction rate, as shown in Fig. 1.

The kinetics of each elementary step in the Arrhenius description is related to the frequency of a reaction's attempts and the ultimate outcome, which is determined by the ability to overcome the barrier. Consequently, the kinetic is not a representation of the timescale of the reaction event, which is more accurately reflected by the time required for atomic and molecular motions related to bond formation and breakage. The latter typically occurs on a timescale ranging from hundreds of femtoseconds (fs) to a few picoseconds (ps)^1^ and is often referred to as dynamics in contrast to kinetics. The result of an elementary step can either lead to the formation of a transient species with a brief residence time, which readily reacts to produce products in the subsequent step, or, in some instances, a long-lived intermediate state that becomes a reaction sink.

The catalytic transformation from reactants to products, as illustrated in Fig. 1, is a rare stochastic event (a typical turnover frequency of an active site is 1 per second). Consequently, if the various species on the surface are monitored using *in situ* techniques under steady-state conditions, only the adsorbed reactants, products, and potentially any stable intermediates can be observed. However, other significant intermediates will only be present transiently, resulting in an overall concentration below the detection limit under steady-state conditions. The use of an ultrashort (femtosecond) optical laser pulse to drive reactions in heterogeneous catalysis provides a new window into these processes. By reaching these ultrashort time scales, it is possible to greatly increase the population of transient intermediates, thereby facilitating their detection on an ultrafast timescale.^1–3^

**FIG. 2.**
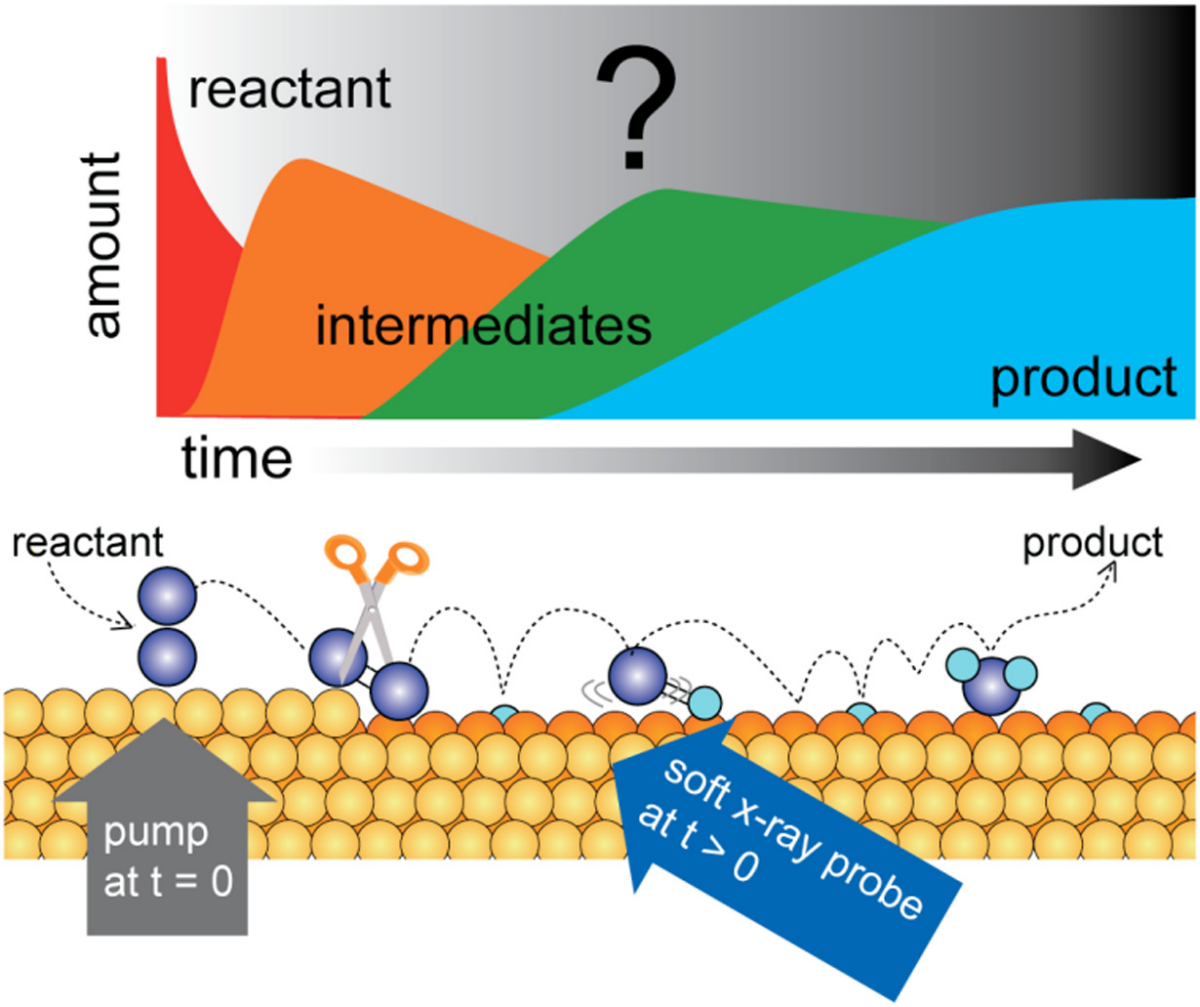
Schematic picture of the elementary steps in a catalytic reaction involving molecular oxygen and hydrogen. The reaction is initiated by an optical laser pump and probed with an XFEL soft x-ray pulse. The concentration of reactants, various intermediates, and products can be followed in real time. Reprinted with permission from Nilsson *et al.*, Chem. Phys. Lett. **675**, 145–173 (2017). Copyright 2017 Elsevier.^3^

Figure 2 illustrates the utilization of an optical, ultrashort laser pulse to increase the population of reactive species that will be transformed into transient intermediates, thereby facilitating detection on an ultrafast timescale.^1–7^ At metal surfaces, ultrashort laser pulses interact with the metal substrate, exciting electrons that subsequently thermalize on a timescale of approximately 100 fs.^8^ These electrons then couple directly to the adsorbate system or via phonons to initiate a reaction.^2^ The heating of the lattice is a slower process than that of the electrons. Consequently, during the initial ps following laser irradiation, a significant non-equilibrium state can be exploited to ascertain reaction mechanisms. The dynamics that are most efficient during the first ps are likely driven by hot thermalized electrons, whereas slower dynamics can also be phonon-driven. Once the reaction has been initiated on an ultrafast timescale, the system can then be probed using spectroscopy techniques with fs pulses, as illustrated in Fig. 2. X-ray Absorption Spectroscopy (XAS) and x-ray Emission Spectroscopy (XES) or sometime denoted Resonant Inelastic x-ray Scattering (RIXS) enables the detection of modifications in the electronic structure and surface chemical bonding as a reaction progresses.^3^ The ability to access ultrafast time scales with these techniques has been made possible by the advent of x-ray free electron lasers (XFELs). The potential of this approach has been firmly established by initial experiments conducted at the Linac Coherent Light Source (LCLS) at SLAC National Accelerator Laboratory and the Free Electron laser Radiation for Multidisciplinary Investigations (FERMI) facility at Elettra Sincrotrone Trieste, wherein pump-probe measurements were employed to investigate fs dynamics in surface chemical reactions.^3,9–21^

Jo Stöhr has pioneered the development of XAS applied to surface adsorbates, resulting in the seminal textbook “NEXAFS Spectroscopy.”^22^ In this paper, I will highlight the usefulness of XAS based on the principles put forward by Jo Stöhr in his textbook and show how the concepts he derived provide insights into the chemical reactivity of surfaces using x-ray lasers. In particular, the polarization dependence of XAS resonance intensities provides insight into the ultrafast reorientation of molecular adsorbates, the chemical shifts of XA resonances allow for the determination of various time-dependent species, the concept of “bond length with a ruler” demonstrates how the intermolecular bond length can be extracted from XAS measurements, and the changes in populations detected with XAS and XES around the Fermi level illustrate how holes and electrons are generated upon laser excitations.

In the following, I will describe the time-dependent changes observed in XAS and XES spectra following laser pumping of atomic carbon on Ni(100). These changes were observed to result from electronic excitations that eventually lead to nuclear motion.^18^ Additionally, XAS of CO adsorbed on Ru(0001) was studied to probe ultrafast vibrational excitation,^13^ desorption,^11,20^ oxidation,^17^ and hydrogenation.^15^ The ability to discern both the polarization dependent intensity and energy position of the π^*^-resonance in XAS is of paramount importance in probing CO as first outlined by Jo Stöhr.^22^

## II. ULTRAFAST ELECTRONIC EXCITATIONS IN SIMPLE ATOMIC ADSORBATES

The dynamical processes of adsorbates on surfaces following laser excitation typically occur in the fs regime. Optical pump-probe experiments have been employed to track the dynamics of adsorbates.^2^ The optical pump excites high-energy electron–hole (e–h) pairs in the metal, which locally thermalize within approximately 100 fs to create a quasi-equilibrium state. This state is often described by a two-temperature model (2T), with one temperature corresponding to the e–h pairs and the other to the substrate phonons. The equilibration between the two systems occurs on a timescale of several ps. The elevated electron and phonon temperatures stimulate vibrational activity in adsorbates, thereby triggering surface chemical reactions. The electronic structure changes of adsorbates due to optical laser excitation of the substrate metal have only been observed indirectly, based on the surface chemistry that is induced.^2^ Until the advent of XFELs, direct experimental probing of the electronic structure changes due to laser excitation on the adsorbate atoms has been lacking.

### A. The d-band model

**FIG. 3.**
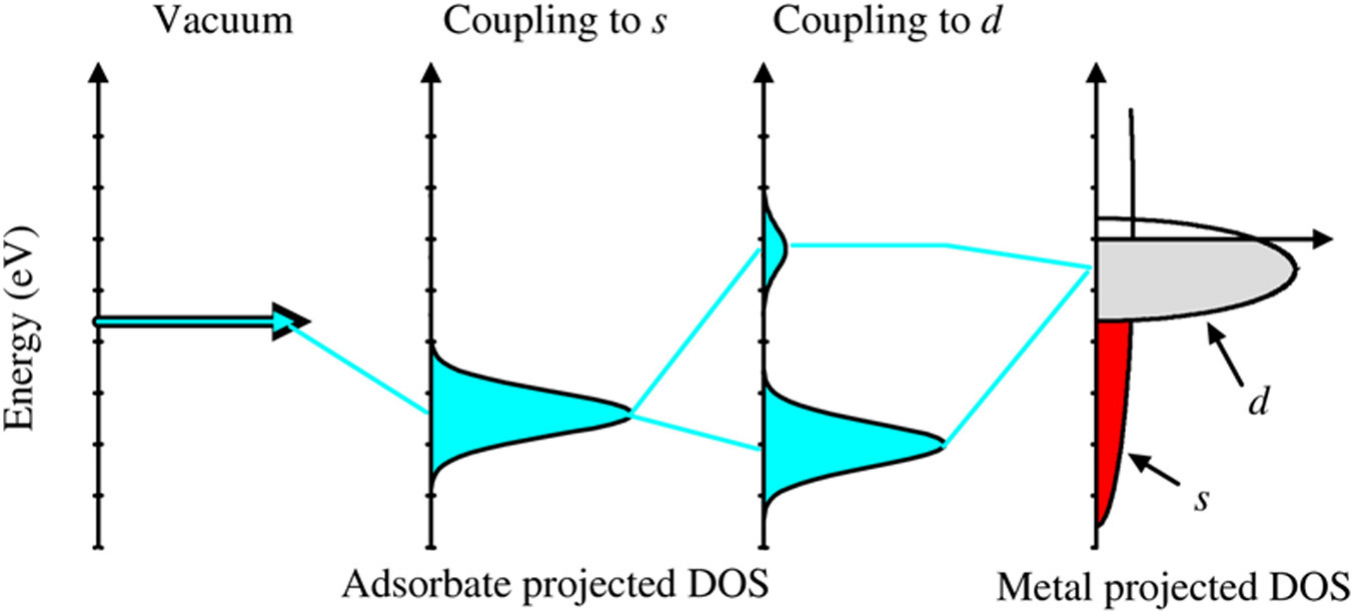
Schematic illustration of the formation of a chemical bond between an adsorbate valence level and the s- and d-states of a transition metal surface. Reprinted with permission from Nilsson *et al.*, Catal. Lett. **100**(3–4), 111–114 (2005). Copyright 2005 Springer Nature.^23^

Before addressing electronic excitations in atomic adsorbates, it is useful to review a simple description of the electronic structure, and how x-ray spectroscopy can provide an atom specific view. The formation of the new adsorbate states can be understood using the d-band model,^23^ which has proven particularly useful in describing bond formation and trends in reactivity among transition metals. Figure 3 depicts a schematic illustration of the electron density of states projected onto the adsorbed atom for two distinct cases.^23^ In this model, the interaction strength between a specific electronic state of the adsorbate and the metal states is often referred to as the hopping matrix element. In the event that the hopping matrix element is considerably smaller than the bandwidth of the metal states, such as the s-electrons of the metal, the interaction results in a broadened resonance level of the projected states on the adsorbate. When the bandwidth is significantly smaller than the hopping matrix element, such as the d-bands, the bonding and antibonding states become distinct electronic levels located below and above the free adsorbate level and the metal d-band, as shown in Fig. 3. The strength of the bond formed between the atoms is contingent upon the relative occupancy of the bonding and antibonding states.

**FIG. 4.**
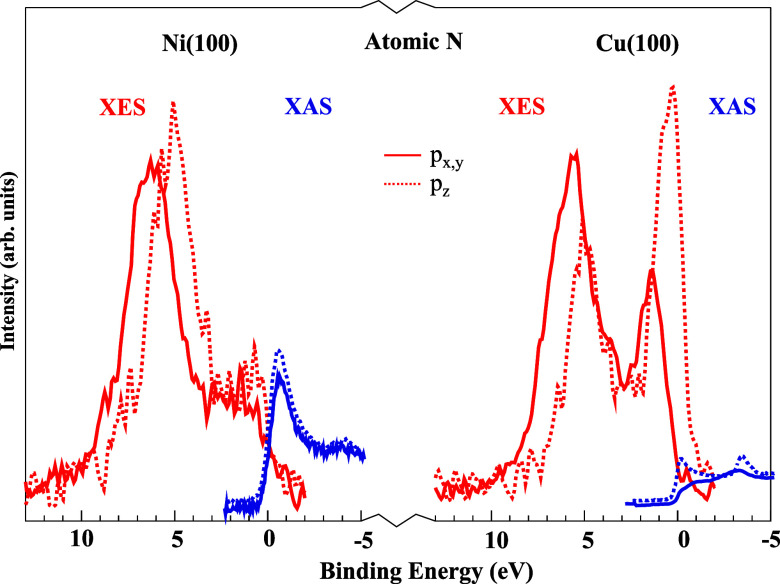
Comparison of the XES (occupied states) and XAS (unoccupied states) spectra of atomic N adsorbed on Ni(100) and Cu(100) with separated *p* components. The intensity scaling between the XES and XAS spectra is arbitrary. Reprinted with permission from Nilsson *et al.*, Catal. Lett. **100**(3–4), 111–114 (2005). Copyright 2005 Springer Nature.^23^

We will illustrate this bonding case with chemisorbed atomic nitrogen, where the distinction in the population of the antibonding states between transition and noble metals, such as nickel and copper, is distinctly evident. Figure 4 shows the XES and XAS spectra on a unified binding energy (BE) scale.^22,24^ The spectra pertaining to the adsorbate (in-plane) p_xy_ states are represented by solid lines, whereas the spectra associated with the (out-of-plane) p_z_ states are illustrated by dotted lines. In the XES spectra of N adsorbed on Cu, both the p_xy_ and p_z_ components exhibit two strong peaks, as seen in Fig. 4. These peaks represent the bonding and antibonding states, respectively. In contrast, the XAS spectra do not display any pronounced peaks. In the case of nitrogen adsorbed on nickel, only one strong peak is observed at high BE in the XES spectra, which can be attributed to occupied bonding states. The antibonding states can be observed in the XES as a plateau close to the Fermi level but more clearly in the XAS spectra as part spills over the Fermi level. The experimental data clearly demonstrate that the antibonding states are shifted from completely below to residing around Fermi level as the adsorbate changes from Cu to Ni.

In Ni metal, the Fermi level is situated at the apex of the d-band, whereas in Cu, it is positioned 2 eV above the d-band, resulting in that the antibonding states are fully occupied during adsorption on Cu and partly unoccupied in the case of Ni. For adsorption on Cu, the sole net bonding effect would originate from the remaining 4sp interaction. The impact of populating the antibonding states is evident in the adsorption energies, which are significantly lower for Cu in comparison to Ni.^23,24^

### B. Ultrafast electronic excitation of chemisorbed C on Ni

To gain insight into the manner by which initial laser excitation modifies the electronic structure of adsorbates, which ultimately gives rise to atomic motion on the surface and is a prerequisite for a surface chemical reaction, the adsorbate carbide overlayer on Ni(100) was subjected to investigation through the use of XAS and XES at FERMI.^18^ This system serves as a prototypical example, wherein the electronic structure of a simple atomic adsorbate is well understood based on the d-band model, which has been successfully probed with x-ray spectroscopy.^24,25^

**FIG. 5.**
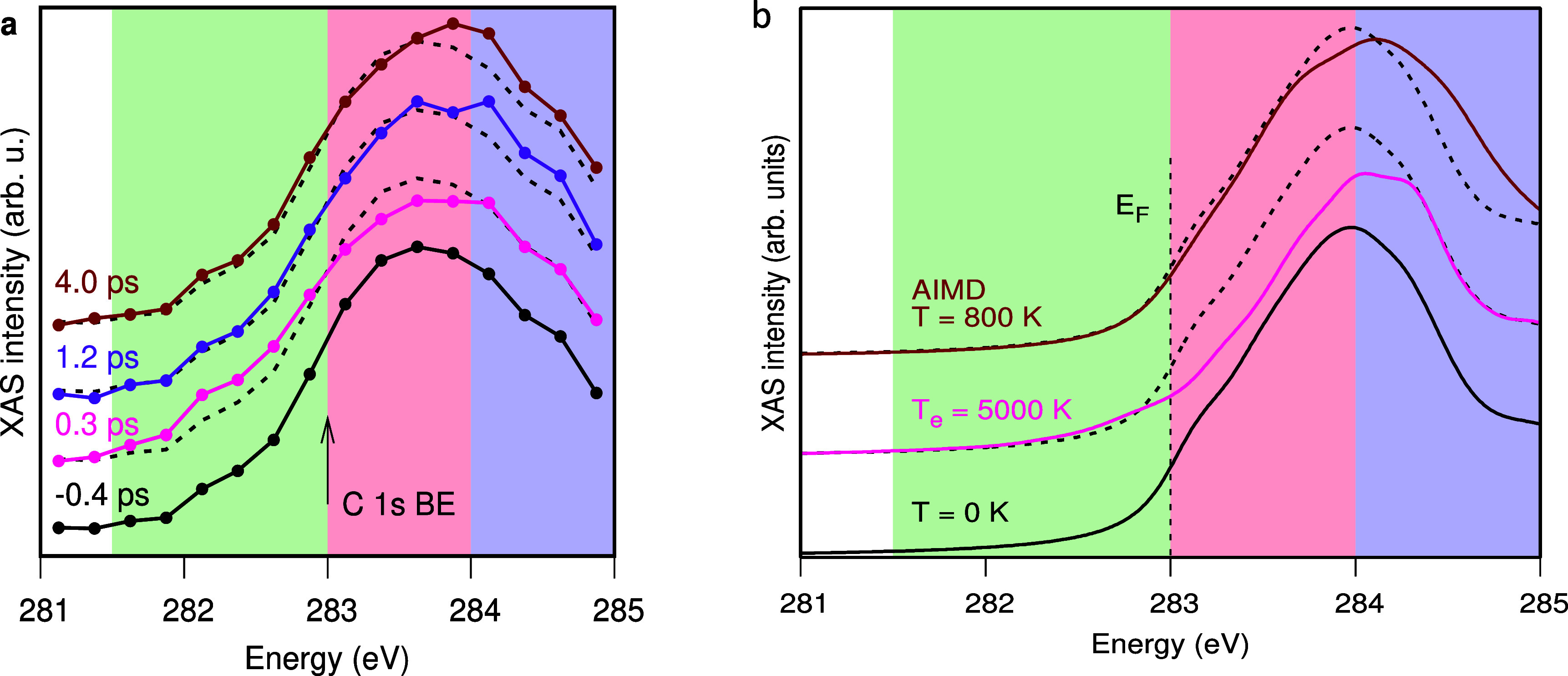
(a) Experimental XAS spectra measured with the E-vector parallel to the surface at different delay times between the optical and soft x-ray laser beams. The arrow indicates the C 1s binding energy used for determining the Fermi level. (b) Computed XAS spectra. The black lines show the vibrational ground state spectra; the brown line shows the result from an *ab initio* molecular dynamics (AIMD) simulation at 800 K; and the pink line shows the result of a high electronic temperature. Reprinted with permission from Schreck *et al.*, Phys. Rev. Lett. **129**, 276001 (2022). Copyright 2022 American Physical Society.^18^

Figure 5(a) shows the C K-edge XAS at varying delay times for C adsorbed on Ni(100) in the p4g (2 × 2) structure following 400 nm excitation.^18^ The laser excitation results in the creation of an electron–hole pair across the occupied and unoccupied parts of the Ni 3d band. This subsequently decays into a hot thermalized electron distribution, which can affect the adsorbate. The XAS probing involves excitation into the antibonding state within the d-band model.^23^ The C1s XAS spectrum at a delay of 0.3 ps exhibits two notable changes: an elevated intensity in the green region below 283 eV and a diminished intensity in the red region above 283 eV [Fig. 5(a)]. The pumped induced changes are less discernible in the spectrum at and above 1.2 ps delay, which instead exhibits an increase in intensity in the region between 284 and 285 eV (blue region).

To facilitate the interpretation of the experimental data, a series of spectral calculations were conducted, encompassing both electronic excitation and nuclear motion.^18^ Figure 5(b) depicts the calculated XAS from the ground state and excited systems, corresponding to a high electronic temperature (Te = 5000 K) and a fully thermalized system at T = 800 K. The theoretical spectra exhibit a qualitative reproduction of the experimental XAS at early times, including the appearance of intensity below E_F_ and a decreased intensity above E_F_. This is achieved by considering the hot electron–hole (e–h) pair distribution on the carbon atom due to laser excitation. The results demonstrate that the thermalized hot electron–hole pair distribution initially created in the Ni lattice is immediately felt by the C adsorbate, resulting in a thermalized electron distribution between the adsorbate and Ni lattice seen in the antibonding resonance. The increase on the high-energy side of the main XAS peak at 4 ps delay is reproduced in the simulation of the spectrum from a fully thermalized system at 800 K. The increasing intensity in this region is consistent with thermal motion of C atoms toward lower coordinated bonding geometries than the fourfold hollow sites they occupy initially.

**FIG. 6.**
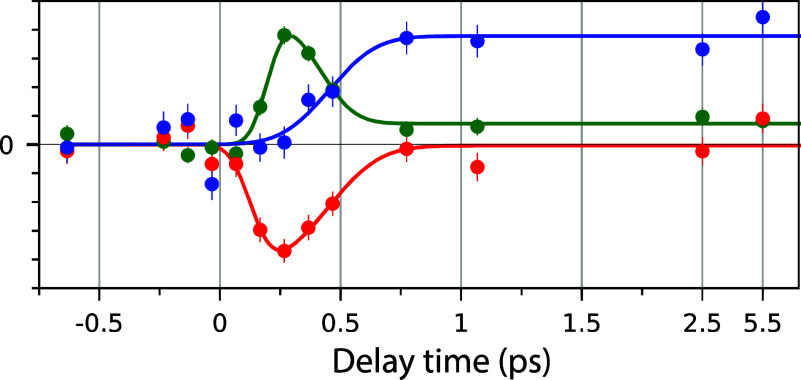
XAS spectral regional intensity of different delay times. The color of each time trace represents different spectral regions indicated in Fig. 11(a). Reprinted with permission from Schreck *et al.*, Phys. Rev. Lett. **129**, 276001 (2022). Copyright 2022 American Physical Society.^18^

Figure 6 illustrates the time traces of the spectral changes depicted in Fig. 5(a).^18^ It is observed that the excited electron–hole population, as seen in the experimental XAS, decays on a timescale of approximately 300 fs, concomitant with an increase in intensity of approximately 1 eV above E_F_. The XAS high-energy shoulder at longer delays is a definitive indicator of a high overall temperature, and its gradual build-up indicates equilibration of the system, which occurs over several ps. We identify this timescale as that of phonon excitation and the response of all substrate degrees of freedom to the excitation pulse.

These spectral changes are consistent with a decreasing electronic temperature via electron–phonon coupling and corresponding heating of the phonon modes and are in good agreement with the time scales of the 2T model for Ni. These two different time regimes are essential to understand how laser excitations can initiate surface chemical reactions by hot thermalized electrons that may change the bonding character at early times (∼100th fs) due to weakening of the bonding character within the d-band model and phonons at later times (ps).^1–3^

## III. LASER INDUCED EXCITATIONS IN ADSORBED CO

Having previously examined the processes involved in a simple atomic adsorbate, we have now turned our attention to a molecular system. For decades, adsorbed CO has served as a prototype system for a well-oriented molecular system, and the nature of the chemical bond has been described in detail.^24,26^ The question, thus, arises as to what ultrafast electronic and vibrational effects can be observed, and how these lead to desorption or reaction with another adsorbate. To address these questions, we have opened the possibility of altering the incoming x-ray polarization vector in ultrafast studies, thereby enabling us to track the impact of specific symmetry orbitals in accordance with the selection rules in XAS for an oriented system.

### A. Ultrafast laser induced vibrational excitation of CO on Ru

The underlying physical processes governing chemical reactions at the surface are the vibrational, rotational, and translational motions of adsorbates.^1–3^ In particular, the manner in which energy is transferred from the bulk substrate to the adsorbate modes can be pivotal for understanding selectivity in catalysis. To address the early time dynamics (below 1 ps) in adsorbed CO on Ru(0001), the FERMI facility provides an additional capability in the form of a seeded x-ray laser with more precise control between pump and probe pulses, thereby enabling an overall time resolution of approximately 100 fs.^13^

**FIG. 7.**
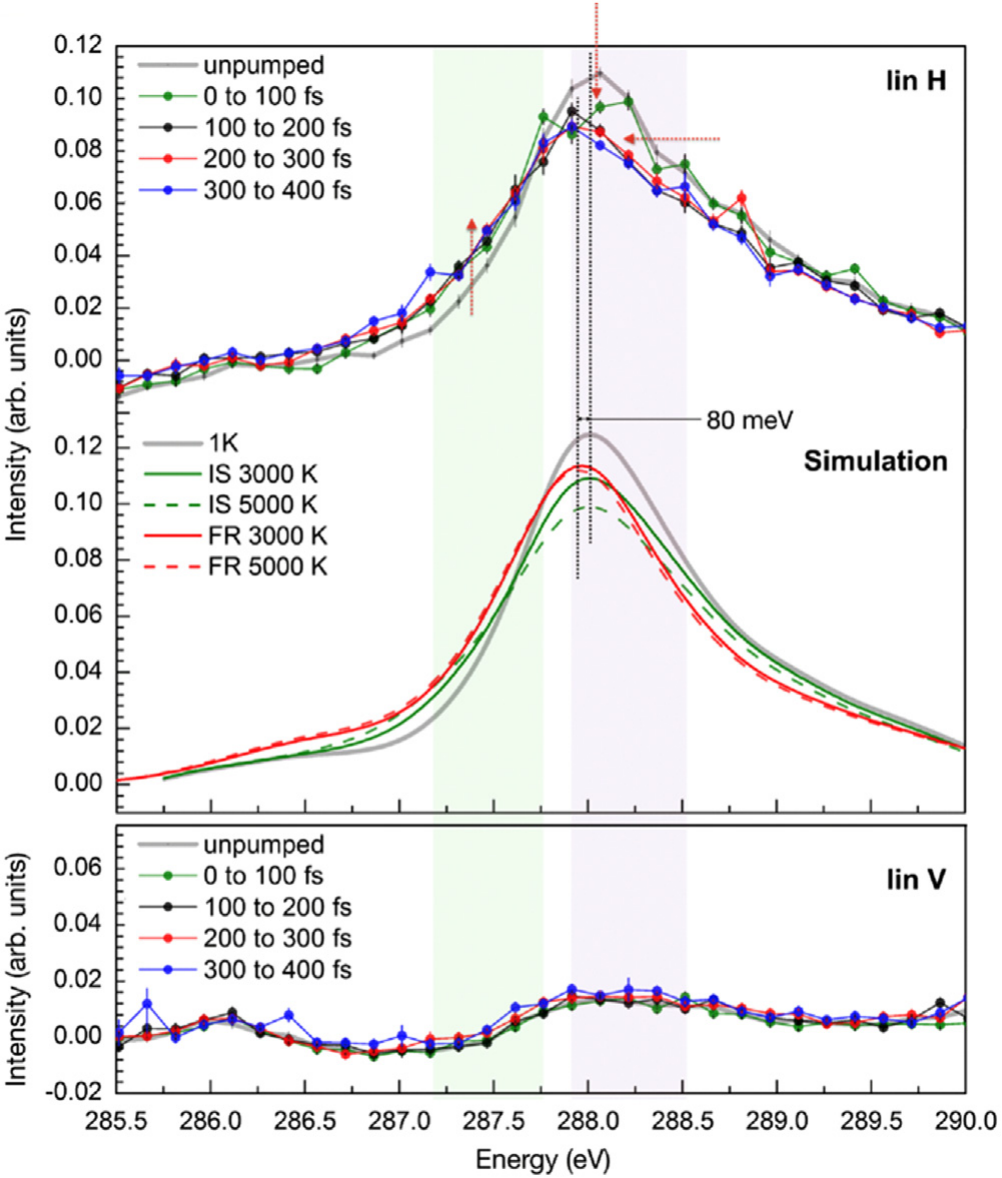
(Top) Upper panel shows time-resolved C1s XAS spectra of CO on Ru(0001) measured with x-ray polarization in-plane to the surface (lin H). Middle panel shows computed C1s XAS with excited internal stretch (IS) and frustrated rotation (FR) modes. Lower panel shows time-resolved C1s XAS spectra with x-ray polarization out-of-plane to the surface (lin V). Reprinted with permission from Diesen *et al.*, Phys. Rev. Lett. **127**, 016802 (2021). Copyright 2023 American Physical Society.^13^

Figure 7 depicts the evolution of the linear in-plane polarized (lin H) C 1s XAS spectra as a function of pump-probe delay time.^13^ Given that CO chemisorbs on Ru(0001) in an upright geometry at the on-top site (with the C atom coordinated toward the Ru atom),^27^ the dipole selection rule indicates that the strongest moment for the C 1s → 2π^*^ transition is observed when the electric field vector (E vector) is parallel to the surface in horizontal polarization. As illustrated in the unpumped spectrum depicted in Fig. 7, a prominent resonance peak centered at approximately 288 eV is observed, which is attributed to the C 1s → 2π^*^ transition. In the bottom panel, the electric field vector is perpendicular to the π-system for out-of-plane polarization (lin V), which results in a markedly diminished intensity. A closer examination of the H-polarized spectra reveals a sudden decrease in intensity at the peak center and an increase in intensity on the lower energy side of the spectrum for delays between 0 and 100 fs, following the laser pump pulse. Nevertheless, the primary peak energy remains unaltered throughout this interval. On the other hand, a redshift of approximately 80 meV of the peak center can be identified in the subsequent 100 fs delay. No further rapid changes are identified in the subsequent delays, indicating that the energy equilibration process occurs at a slower rate after the initial excitation. The bottom panel displays the lin V data, which indicates that the decrease in intensity in the lin H spectrum at the 0–100 fs delay cannot be attributed to the molecule tilting since no corresponding intensity increase at 288 eV can be observed in the lin V spectrum. Instead, a gradual increase in overall intensity in the lin V spectra can be seen in the subsequent delays, up to approximately 1 ps. The decrease instead needs to be due to changes in the electronic structure that affects the 2π^*^ population on the CO molecule via vibrational excitations.

**FIG. 8.**
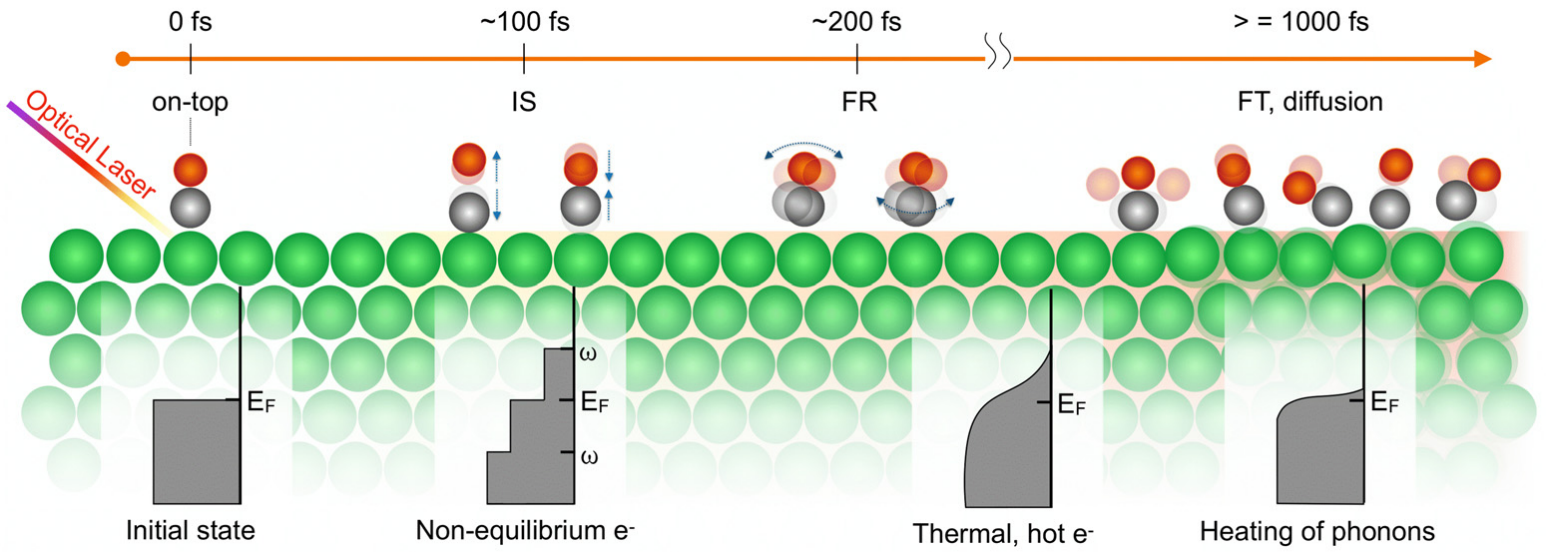
Sketch of the system evolution during the first picosecond. Atom colors: O red, C gray, and Ru green. Reprinted with permission from Diesen *et al.*, Phys. Rev. Lett. **127**, 016802 (2021). Copyright 2023 American Physical Society.^13^

To interpret the time evolution of the spectra, we performed DFT spectral calculations, which are shown in the middle panel of Fig. 7, and the spectral changes are well reproduced.^13^ In summary, the time-dependent process, as illustrated in Fig. 8, begins with an initial excitation of the CO internal stretch mode (IS) within 100 fs of the laser pulse, followed by excitation of the frustrated rotation (FR) mode during the subsequent 100 fs. The thermalization of the substrate phonons and heating of the adsorbate translational modes occur over the first ps, consistent with the commonly used 2T model. The resulting picture is that high excitation of the internal stretch and frustrated rotational modes occur within 200 fs of laser excitation, followed by thermalization of the system in the ps regime. The initial excitations are significantly faster than what can be accounted for in nonadiabatic friction models with theoretically obtained friction coefficients. This indicates a lack of current theoretical descriptions of the coupling between highly excited electron distributions and adsorbate dynamics.

### B. CO desorption probed with polarization sensitive XAS

The existence of a short-lived and weakly bonded precursor state that could reside on the surface prior to desorption has been a topic of considerable debate for an extended period of time.^28–31^ This transient precursor state is typically regarded as an intermediate state that facilitates the regaining of rotational and translational energy in the adsorbed molecule prior to the complete rupture of the adsorption bond with the surface, resulting in its desorption into the gas phase. The presence of CO in this transient precursor state has been directly confirmed using both LCLS at the O 1s XAS and XES spectra^11^ and FERMI through the utilization of polarization dependent experiments at the C-edge.^20^ Here, we present the findings from the latter, which offer a more comprehensive insight into the precursor state.

**FIG. 9.**
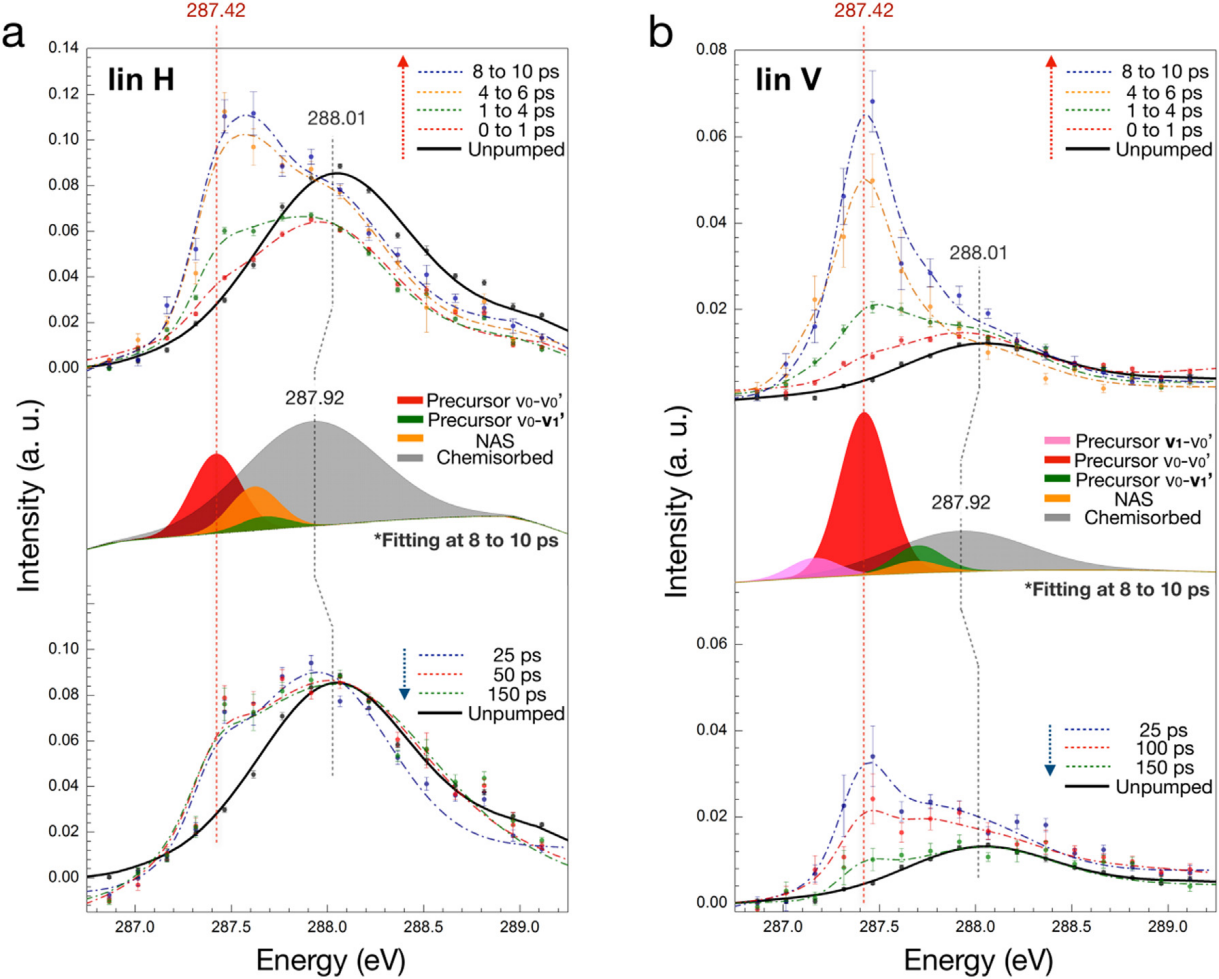
Time-resolved XAS spectra of chemisorbed CO on Ru (0001) on longer time scales following excitation by 400 nm laser excitation by (**a**) lin H (in-plane polarization with respect to the surface) and (**b**) lin V (out-of-plane polarization) x-ray pulses. The top panel shows the pump–probe spectra evolving from negative delay (unpumped) to +10 ps. The middle panel shows the decomposed spectral distribution of characteristic states for the spectrum at 8–10 ps delay. Most of the spectral features slowly recover to their unpumped state at the longer delay. The vertical line at 287.42 eV indicates the position of the gas phase π^*^ resonance. Reproduced with permission from Wang *et al.*, Phys. Chem. Chem. Phys. **22**, 2677–2684 (2020). Copyright 2020 Royal Society of Chemistry.^20^

Figure 9 depicts the in-plane (lin H) and out-of-plane (lin V) C K-edge XAS spectra of the C 1s-to-π^*^ region as a function of pump-probe delay time.^20^ In the lin H spectra, a decrease in the chemisorbed component at 288 eV is observed, accompanied by the emergence of a new feature around 287.4 eV. The former, which is pronounced in the lin H spectra, corresponds to chemisorbed CO in the standing-up geometry, as discussed previously. The latter is situated in close proximity to the 2π^*^ resonance gas phase value and predominantly discernible in the lin V spectra. It is attributed to the precursor state in the context of desorption where also flat-lying geometry is populated in almost free rotation. The population of the precursor state reaches its maximum at 8–10 ps and subsequently exhibits a gradual decline at longer timescales as the chemisorbed state regains intensity.

**FIG. 10.**
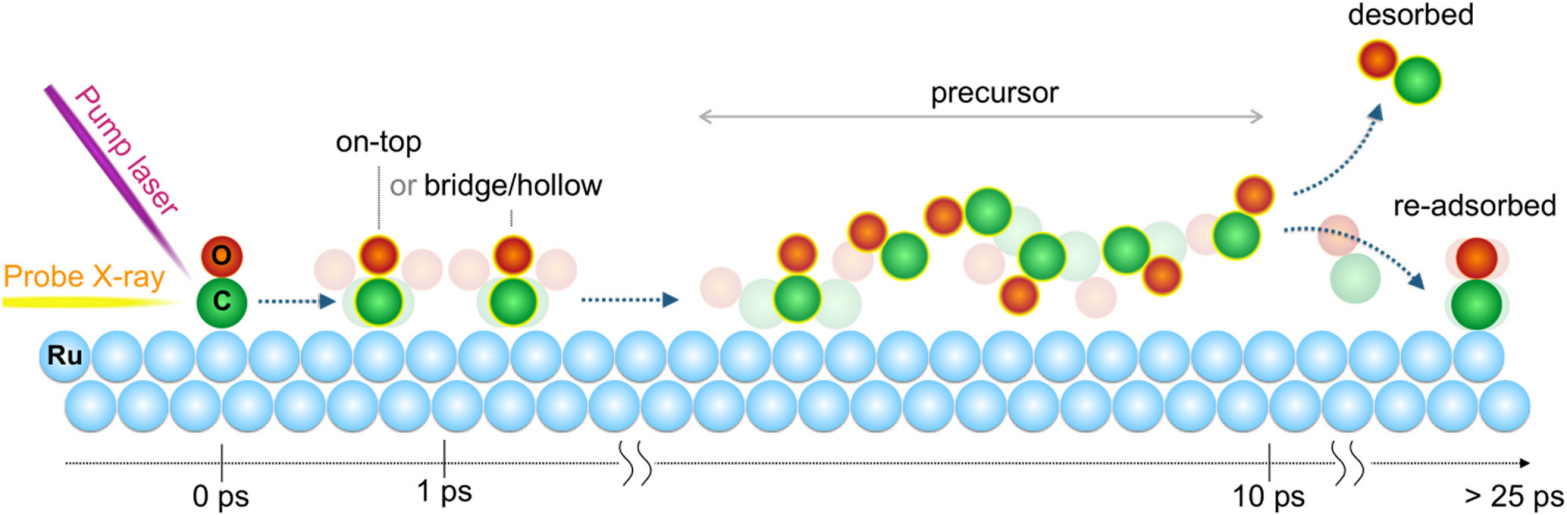
Dynamic evolution as a function of pump–probe delay for CO chemisorbed on Ru(0001) following optical laser excitation. After the laser irradiation on Ru(0001), CO molecules are shaken and diffuse from the on-top sites toward more highly coordinated sites, followed by isotropically rotating precursor state CO populating the surface. A fraction of CO in the precursor state proceeds to desorption, while the majority re-adsorb back onto the surface. Reproduced with permission from Wang *et al.*, Phys. Chem. Chem. Phys. **22**, 2677–2684 (2020). Copyright 2020 Royal Society of Chemistry.^20^

Our findings have enabled the construction of a comprehensive representation of the process and its constituent steps,^20^ as illustrated in Fig. 10. As previously discussed, the arrival of the pump laser pulses results in the sudden excitation of chemisorbed CO molecules situated at on-top sites. These molecules become rotationally and translationally hot, diffusing toward highly coordinated sites with a wobbling molecular motion during the first ps. Once a portion of the excited adsorbates have successfully crossed the entropy barrier that separates the chemisorbed and precursor states, the isotropically rotating CO molecules in the precursor state repopulate the surface over time. It is estimated that approximately 14% of the CO molecules in the precursor state ultimately break away from the surface and enter the gas phase. This estimation is based on the lin V XA spectra, as they are selectively sensitive to the precursor state. This is due to the fact that the C 1s → π^*^ transition with vertical polarization of the chemisorbed CO in the standing up geometry is dipole forbidden.

It is interesting to note that for the co-adsorbed CO plus O system that will be described subsequently with regard to the oxidation reaction, the CO desorption channel does not occur through the precursor state. Instead, it occurs via another mechanism that was discovered from a pump-probe XAS study conducted at LCLS.^21^ The modification of the electronic structure in the metal due to the presence of adsorbed O atoms results in a change to the potential energy surface of adsorbed CO resulting in a reduction in the free energy barrier. This modification facilitates a more direct CO desorption to the gas phase, preventing trapping in a weakly adsorbed state.

## IV. LASER INDUCED ULTRAFAST CO SURFACE CHEMISTRY

A number of catalytic reactions entail CO as a reactant. In automobiles, the exhaust gas is cleaned by oxidizing the poisonous CO to CO_2_ using noble metal catalysts. For the large-scale chemical production of synthetic fuels and base chemicals, catalytic CO hydrogenation is an essential step. In this context, x-ray lasers-based XAS has proven invaluable for detecting species in close proximity to the transition state as well as short-lived transient intermediate species, during laser induced CO oxidation and hydrogenation catalytic reactions on Ru(0001).^15,17^

### A. Observing transient species close to the transition state in CO oxidation

Molecules at the transition state (TS) are exceedingly difficult to capture or observe due to the near-zero instantaneous population at steady-state conditions. However, TS is the key to understanding chemical reactivity.^32^ It separates reactants from products, and the free energy required to reach this point determines the kinetics of an elementary chemical reaction. The use of ultrafast pump-probe techniques provides opportunities for probing molecular structures in the vicinity of the TS region, provided that the potential energy surface is sufficiently flat. The laser excitation allows for the promotion of a significant population of molecules, thus enabling detection on short time scales.^32,33^

We have demonstrated that ultrafast pump-probe XAS with the XFEL LCLS can be used to probe the electronic structure of molecular species in the TS region during CO oxidation on a Ru(0001) surface.^17^ A 400 nm optical laser pump pulse was employed to excite the electrons in the substrate, as previously described in IIA. Subsequently, energy transfer to the adsorbate system resulted in a rapid increase in adsorbate-substrate vibrational excitations, which then facilitated the CO oxidation reaction on ultrafast timescales. In this instance, the O K-edge XAS was employed to monitor the temporal evolution of the unoccupied valence electronic structure in the vicinity of the adsorbed O and CO species throughout the course of the oxidation process. The advantage of this approach is that the O and CO are co-adsorbed on the Ru (0001) surface, creating reactive units in close proximity. Upon laser excitation, these units can begin to collide. The spectroscopic contrast was interpreted based on DFT calculations of specific species identified from free energy calculations along the reaction pathway.^17^

**FIG. 11.**
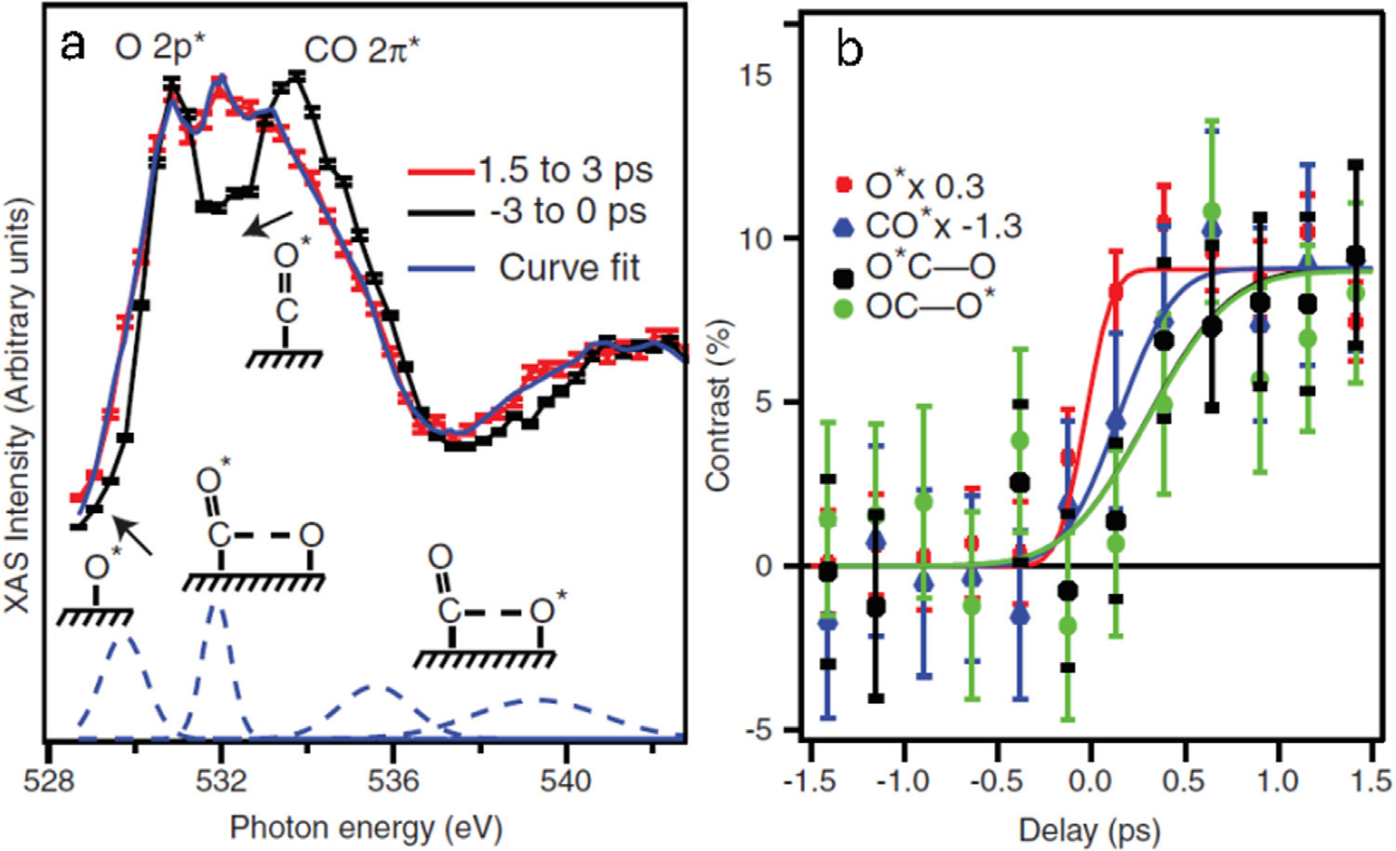
Measured x-ray absorption spectra with time-dependent changes. (a) Pump-probe O K-edge XAS spectrum of CO/O/Ru(0001). (b) Time development of the spectral intensities in four different spectral regions. Reprinted with permission from Öström *et al.*, Science **347**, 978–982 (2015). Copyright 2015 AAAS.^17^

Figure 11(a) depicts the O K-edge XAS spectra for negative time delays and at 1.5–3.0 ps post-laser excitation, whereas Fig. 11(b) illustrates the time evolution of the significant spectral features shown in Fig. 11(a).^17^ The rapid response observed in the shoulder between 528 and 529 eV is indicative of the activation of the O species on the surface. The increased intensity emerges on a timescale of 280 ± 100 fs, which is commensurate with the experimental time resolution. The O species couple strongly to the electron bath, where hot electrons from the surface transfer transiently to the O-Ru antibonding orbital, as was demonstrated in the case of C on Ni^18^ in Sec. II B, and the O-Ru bond distance is now elongated from the equilibrium value. This increased bond distance is manifested as kinetic energy in the form of nuclear motions of the O surface atoms as the transiently excited hot electrons delocalize back into the conduction band. The O atoms, which typically occupy the hollow site on Ru(0001), gain sufficient kinetic energy to populate less coordinated sites, such as bridge sites. This results in a shift toward lower energy in the XAS spectra, as seen in Fig. 11(a). The activated O atoms with reduced bond order to the surface can now more readily interact with the surface CO species.

The CO molecules on the surface are also activated, though the timescale for this process is slightly longer, at 550 ± 120 fs.^17^ This phenomenon is evidenced by the redshift of the CO 2π^*^ peak, as illustrated in Fig. 11(a). As previously outlined in Sec. III A, the activation of CO molecules into frustrated rotation is a key phenomenon. The frustrated rotation increases the translational motion of CO on the surface, allowing the CO molecules to move to more highly coordinated sites, such as bridge and/or hollow sites. The enhanced mobility of both surface species gives rise to heightened interaction between CO and O, which, in a subset of instances, culminates in the formation of transient OC–O bonds (800 ± 250 fs).

This novel bond between CO and O is evidenced by the emergence of new states within the energy range of 536–539 eV and around 532 eV. The former region corresponds to probing a σ^*^ OC–O antibonding orbital, where the bond length between the CO and O atom is determined by the concept of bond length with a ruler^22^ to be between 1.4 and 1.6 Å.^17^ This bond length is considerably longer than that observed in CO_2_ (1.2 Å). The resonance at 532 eV is attributed to the O atom in the CO molecule, with the adsorption site shifting from on-top to hollow due to interaction with the reacting O atom.^17^ The presence of both resonances indicates the existence of populated species in the region close to the transition state.

**FIG. 12.**
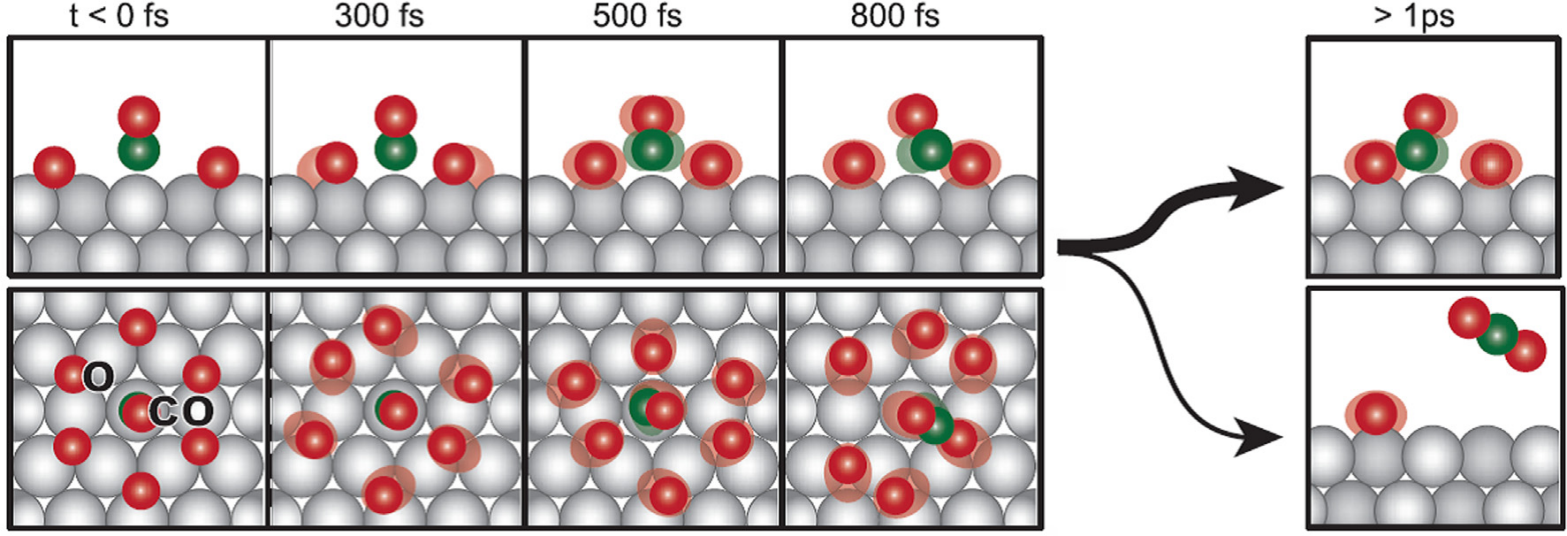
Schematic of the different steps in the laser-induced CO oxidation on Ru(0001), including relevant timescales. Reprinted with permission from Öström *et al.*, Science **347**, 978–982 (2015). Copyright 2015 AAAS.^17^

Based on the pump-probe observations, we present a summary of the findings in the form of a simple illustration, as shown in Fig. 12.^17^ The O atom becomes activated with motions parallel to the surface on a timescale below 300 fs, whereas the CO atom is activated on a timescale of 500 fs. The CO molecules will begin to collide with O on the surface, as they are constrained by the O atoms in neighboring sites within the co-adsorbed structure. These collisions result in the generation of a transient population of species in the vicinity of the TS region, which emerges on timescales slightly longer than the initial motion of the CO and O species. The majority of collisions result in the dissociation of CO and O, followed by further collisions into TS. Only a minor proportion of events lead to the formation of CO_2_; this is in line with expectations, given that both classical and quantum mechanical models indicate that the transition over a potential energy barrier entails a deceleration as kinetic energy is converted to potential energy.^17^

The capacity to investigate transient species in close proximity to the TS offers a comprehensive understanding of the electronic states of reacting molecules at surfaces. This possibility provides a unique benchmark for the theoretical description of surface-catalyzed processes, which is expected to be a crucial tool in advancing our fundamental understanding of surface chemical processes in heterogeneous catalysis.

### B. Short-lived HCO transient intermediate in CO hydrogenation

The hydrogenation of CO represents a pivotal step in the formation of fuels and base chemicals through the use of Fischer–Tropsch (FT) synthesis in syngas utilization and artificial photosynthesis of CO_2_ reduction.^34^ Ru is one of the most effective FT catalysts for the conversion of CO and hydrogen into hydrocarbon chains with high selectivity. One potential hydrogenation elementary step is the addition of adsorbed H to CO on the surface, forming either CHO or COH.^35,36^ However, none of these species has been identified experimentally under normal steady-state reaction conditions.

To identify transient species in CO hydrogenation, we utilized a pump-probe approach with co-adsorbed H and CO on Ru(0001), analogous to the previous example of CO oxidation. We conducted similar probing of the species formed during the hydrogenation of CO using O-K edge XAS spectra at the LCLS XFEL facility.^15^ Prior to the XFEL studies, mass spectroscopy measurement of single femtosecond 400 nm pulses identified desorbing species of a mass corresponding to hydrogenated CO species, where it was not possible to determine whether CHO or COH was generated.^15^ Additionally, a small amount of H_2_CO was detected as a desorbing species based on the mass spectroscopic signal.^15^

**FIG. 13.**
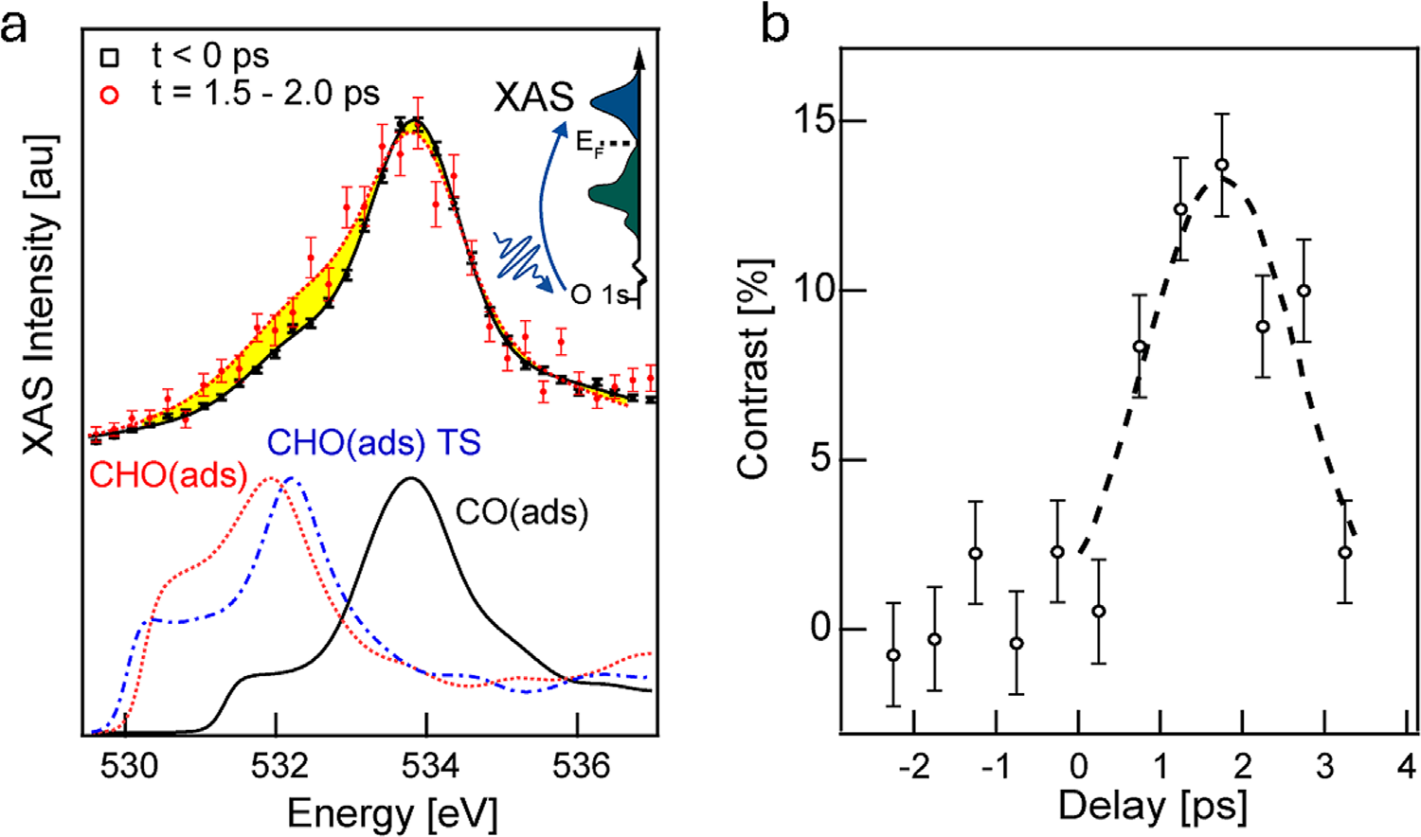
(a) O K-edge XAS at negative delay for the unpumped system and at 1.5–2.0 ps after laser excitation, and simulated spectra for CO(ads) (black, solid), CHO(ads) (red, dot), and the transition state from CO(ads) to CHO(ads) (TS, blue, dash-dot). The inset shows a schematic illustration of the excitation process from the O1*s* level to the unoccupied 2π^*^ resonance in XAS. (b) The intensity contrast in the x-ray absorption spectra integrated between 530.2 and 533.0 eV vs time delay. Reprinted with permission from LaRue *et al.*, J. Phys. Chem. Lett. **8**, 3820–3825 (2017). Copyright 2017 American Chemical Society.^15^

Figure 13(a) shows the O1s XAS for unreacted CO(ads), which was measured at delays where the probe pulse arrived before the pump pulse (t < 0 ps) and for reacting CO(ads), which was probed at a delay time of 1.5–2.0 ps after the reaction was initiated.^15^ The intense peak at 533.8 eV is attributed to the O 1s →2π^*^ electronic excitation of CO(ads) (CO 2π^*^), which is observed in both unreacted CO(ads) and reacting CO(ads). Notably, an additional intensity emerges at lower photon energies as a broad feature around 531.6 eV for reacting CO(ads).

To assist in the interpretation, we used spectral calculations of species along the minimum energy reaction path.^15^ Figure 13(a) shows the transition states during the formation of CHO(ads) as well as CHO (ads) and COH(ads). We can see in Fig. 13(a) that the addition of hydrogen to C in CO shifts the π^*^ resonance down in energy (while the addition to the O atom leads to a significant upwards shift). Considering these trends, the observed transient features in the low-energy region of O1*s* XAS indicate that the reaction is directed toward formation of CHO(ads) or more generally CH_x_O(ads) species. This interpretation is further confirmed by considering XES.^15^

Figure 13(b) illustrates the temporal evolution of the O1s XAS signal, integrated over the energy range of 530.2–533.0 eV.^15^ This evolution corresponds to the intensity of CHxO(ads) species, based on the assignment shown in Fig. 13(a), plotted as a function of the delay between the laser pump and the x-ray probe. The evolution of transient CHxO(ads) species is observable only at short time scales, t < 3 ps. On a longer timescale, CO(ads) transitions to the weakly adsorbed precursor state prior to desorption, as previously discussed in Sec. III B. The quantity of transient CHxO(ads) species attains a maximum value approximately 1.5 ps following the laser pulse, subsequently declining on a timescale of 1.5–2 ps. The rapid rise at t = 0–2 ps and the sudden drop at t = 2–3 ps indicate that CHxO(ads) is formed during the relaxation period of the electronic temperature, whereby the electron bath in the substrate couples strongly to the lateral motions of H(ads) and the frustrated rotation of CO(ads).

The results demonstrate that only CHO species can form during CO hydrogenation reactions, and not COH. However, the residence time for the CHO species is relatively brief, and in FT reactions, only an extremely transient population is feasible. It is probable that other elementary reaction steps will prove to be of greater significance for the FT process.

## V. CONCLUSION AND OUTLOOK

The tremendous progress in time-resolved XAS-based surface chemistry enabled by XFELs and the even more exciting future prospects pay tribute to the pioneering work in the field started by Jo Stöhr over 40 years ago. It is possible to observe transient intermediates that have an extremely short residence time, on the order of a few ps. It also allows the study of a portion of the highly excited potential energy surface near the transition state, a hypothesis that has often been considered implausible.

Finally, a long-standing goal in surface science and catalysis has been to study surfaces under catalysis in real-world conditions. The XAS technique used in this review is based on photon detection, and with a photon-in and photon-out method, the possibility of non-vacuum studies is a real possibility. We can envision the use of a new class of XAS-based instrumentation that allows the study of reactions under near-atmospheric conditions, facilitating the probing of key hydrogenation processes that are critical for the synthesis of ammonia, methanol, and hydrocarbons. The use of water vapor at near-condensation pressures can also enable XAS studies of electrocatalysis, allowing the detection of surface species during fuel cell, water splitting, and electrocatalytic CO_2_ reduction reactions. This prospect involves the integration of ultrafast techniques with operando spectroscopy, allowing the study of surfaces under high pressure conditions and at solid-electrolyte interfaces. Such a technique would allow us to detect transient species as the actual catalytic process occurs as well as the dynamic rearrangement of substrate atoms. Extending this prospect beyond single crystal surfaces to well-defined nanoparticles would be the logical next step. It would allow experimental efforts to keep pace with the rapid development of theoretical simulations. It will be fascinating to watch this field develop over the next decades.

## Data Availability

Data sharing is not applicable to this article as no new data were created or analyzed in this study.
